# Study Designs and Statistical Analyses for Biomarker Research

**DOI:** 10.3390/s120708966

**Published:** 2012-06-29

**Authors:** Masahiko Gosho, Kengo Nagashima, Yasunori Sato

**Affiliations:** 1 Graduate School of Engineering, Tokyo University of Science, 1-3 Kagurazaka, Shinjuku-ku, Tokyo 162-8601, Japan; 2 Faculty of Pharmaceutical Sciences, Josai University, 1-1 Keyakidai, Sakado-shi, Saitama 350-0295, Japan; E-Mail: nagasima@josai.ac.jp; 3 Clinical Research Center, Chiba University of Medicine, 1-8-1 Inohana, Chuo-ku, Chiba 260-8677, Japan; E-Mail: yasu@faculty.chiba-u.jp

**Keywords:** biomarker adaptive design, confounding, multiplicity, predictive factor, statistical test

## Abstract

Biomarkers are becoming increasingly important for streamlining drug discovery and development. In addition, biomarkers are widely expected to be used as a tool for disease diagnosis, personalized medication, and surrogate endpoints in clinical research. In this paper, we highlight several important aspects related to study design and statistical analysis for clinical research incorporating biomarkers. We describe the typical and current study designs for exploring, detecting, and utilizing biomarkers. Furthermore, we introduce statistical issues such as confounding and multiplicity for statistical tests in biomarker research.

## 1. Introduction

In recent years, biomarkers have played an increasingly important role in drug discovery, understanding the mechanism of action of a drug, investigating efficacy and toxicity signals at an early stage of pharmaceutical development, and in identifying patients likely to respond to treatment. In addition, several potentially powerful tools to decipher such intricacies are emerging in various fields of science, and the translation of such knowledge to personalized medicine has been promoted and has occasioned strong expectations from almost every sector of health care. Therefore, biomarkers have been utilized to personalize medication or healthcare and in the safety assessment of drugs in clinical practice. However, few valid biomarkers at present can predict which group of patients will respond positively, which patients are non-responders, and who might experience adverse reactions to the same medication and dose. Therefore, a vast number of clinical biomarker studies are conducted and reported.

In practice, however, the results in highly cited biomarker studies often significantly overestimate their findings, as seen from meta-analyses of these studies. Many of these studies were relatively small and among the first to report on the association of interest. Discoveries made in small studies are prone to overestimating or underestimating the actual association. Ioannidis and Panagiotou [1] revealed that evidence from multiple studies, in particular large investigations, is necessary to appreciate the discriminating ability of these emerging risk factors, and rapid clinical adoption in the absence of such evidence may lead to wasted resources. Biomarker research parallels therapeutic research, with all the same potential biases. Therefore, it is critical in biomarker research to adhere to statistical principles and follow a sound statistical methodology to minimize bias and maximize precision.

In this paper, we first introduce the definition, classification, and some examples of biomarkers in clinical research. Second, we review the typical and current study designs of clinical research using biomarkers in practical studies. Furthermore, we describe statistical issues such as confounding and multiplicity for statistical tests in biomarker research. The final section is a brief summary.

## 2. Definition and Classification of Biomarkers

An expert working group at the National Institutes of Health (NIH) has defined a biological marker or biomarker as ‘a characteristic that is objectively measured and evaluated as an indicator of normal biological processes, pathogenic processes, or pharmacologic responses to a therapeutic intervention’ [2]. According to this definition, biomarkers cover a rather wide range of data types, for example, biochemistry laboratory tests on blood, function testing, electrocardiographic testing, and image information such as computed tomography (CT), magnetic resonance imaging (MRI) and positron-emission tomography (PET). Typical examples of such biomarkers are listed in Table 1 [3–10].

**Figure 1.**
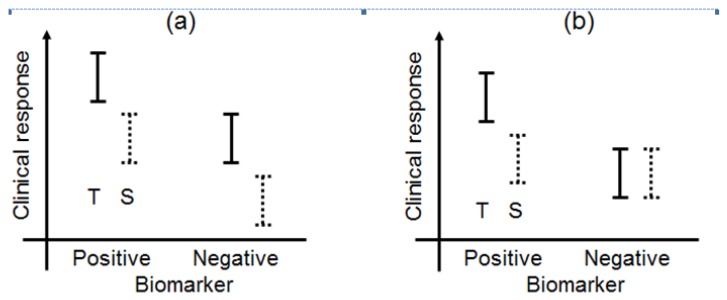

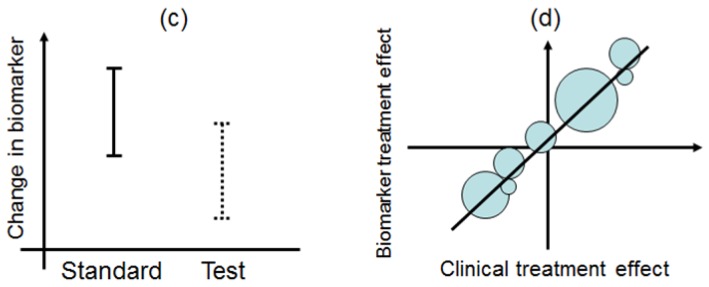
Biomarker types. (**a**) Prognostic biomarker, (**b**) predictive biomarker, (**c**) pharmacodynamic biomarker, (**d**) surrogate endpoint. ‘S’ and ‘T’ denote standard and test treatments, respectively.

Biomarkers can be broadly classified into prognostic biomarkers, predictive biomarkers, pharmacodynamic biomarkers, and surrogate endpoints [5,11]. The biomarker types have been illustrated in a simple manner in Figure 1. In this paper, we have focused on prognostic and predictive biomarkers and have not discussed pharmacodynamic biomarkers or surrogate endpoints in great detail.

### 2.1. Prognostic Biomarkers

A prognostic biomarker classically identifies patients with differing risks of a specific outcome, such as progression or death [12,13]. Recently, the prognostic biomarker was defined as a single trait or signature of traits that separates a population with respect to the outcome of interest, regardless of the types of therapies or treatments [14]. For example, under this definition, if a specified biomarker were prognostic, the outcome (clinical response) of patients with biomarker-positive status would be better than that of patients with biomarker-negative status in both the test and standard treatments. Additionally, the differences of the outcome between test and standard treatments between the biomarker-positive and biomarker-negative populations in Figure 1(a) would be uniform. According to Chakravarty *et al.* [15], a prognostic biomarker is also a baseline patient characteristic independent of therapy or treatment which categorizes patients on the basis of the degree of the outcome.

The prognostic biomarker can distinguish populations into groups whose outcome will be poor or good following the test and standard treatments, but it cannot guide the choice of a particular treatment. The preliminary knowledge necessary to propose a validation study of a prognostic biomarker is the subject of considerable previous work [13]. The current uses of prognostic biomarkers are stated in Table 1.

### 2.2. Predictive Biomarkers

A biomarker predicts the differential outcome of a particular therapy or treatment (e.g., only biomarker-positive patients will respond to the specific treatment or to a greater degree than those who are biomarker negative) [14]. In addition, Chakravarty *et al.* [15] state that a predictive biomarker is a baseline characteristic which categorizes patients by their degree of response to a particular treatment.

In this case, for example, biomarker-positive patients perform moderately better than do biomarker-negative patients when standard treatment is administered, whereas test treatment may be more effective in the biomarker-positive group (Figure 1(b)). As a currently used predictive biomarker, irinotecan-treated patients who were homozygous for the uridine-diphosphoglucuronosyl transferase 1A1 (UGT1A1)*28 allele had a greater risk of hematologic toxic effects than did patients who had one or two copies of the wild-type allele (UGT1A1*1) [16–19]. A diagnostic test for the UGT1A1*28 genotype for irinotecan dosing was approved by the Food and Drug Administration (FDA) in 2005, and the test could be useful for identifying patients with a greater risk of developing irinotecan toxicity. As this example demonstrates, a validated biomarker can prospectively identify patients who are likely to have a favourable clinical outcome from a specific treatment; therefore, a predictive biomarker could guide the choice of treatment in one of several ways.

As a further remark, many biomarkers would have both prognostic and predictive features. For example, in breast cancer, patients with diagnosed estrogen receptor (ER)-negative have a higher risk of relapse than do ER-positive patients with a similar disease stage. In this case, ER is ‘prognostic.’ On the other hand, the antiestrogen tamoxifen is more effective in preventing breast cancer recurrences in ER-positive patients than in ER-negative patients. In this case, ER is ‘predictive’ of benefit from tamoxifen [4]. Now ER is well established as a biomarker that provides prognostic and predictive information as well as a valid target for therapy; therefore trial designs should carefully take such a biomarker into account.

We also notice a usual error in non-randomized studies. A test treatment was administered to biomarker-positive and biomarker-negative patients in a non-randomized study, and the outcome for the biomarker-positive patients was superior to that for the biomarker-negative patients. If the biomarker was prognostic, it might be concluded that biomarker-positive patients would have better outcome regardless of the treatment. On the other hand, if the biomarker was predictive, it might be concluded that biomarker-positive patients would be more likely to benefit from the test treatment. Unfortunately, non-randomized studies cannot provide definitive information to these correct answers.

### 2.3. Pharmacodynamic Biomarkers

According to Jenkins *et al.* [5], when the change in a biomarker is the parameter that is to be understood, explained, or controlled, then the biomarker is considered an endpoint (Figure 1(c)). The biomarker could be used in this sense as a biomarker of the drug activity to demonstrate proof of principle and be used to optimise the dosing schedule of the drug during the earlier phases of the drug development program (*in vitro* studies, animal experiments, and phase I trials), while clinical biomarkers are used in phase II and III clinical trials.

For example, inflammatory markers such as C-reactive protein (CRP) or erythrocyte sedimentation rate (ESR) may be used to select a dose in rheumatoid arthritis treatment or can form part of a clinical composite such as disease activity score (DAS) 28 and be used for the same purpose [5]. Moreover, a recent article reported that carriers of a reduced-function CYP2C19 allele had significantly lower levels of the active metabolite of clopidogrel, diminished platelet inhibition, and a higher rate of major adverse cardiovascular events [7]. Therefore, concomitant proton pump inhibitor use may reduce the formation of the active metabolite and consequently reduce the efficacy of clopidogrel, resulting in more cardiovascular events.

### 2.4. Surrogate Endpoints

A surrogate endpoint is intended to be a substitute for a clinical endpoint. It is expected to predict clinical benefit (lack of benefit or harm) based on epidemiologic, therapeutic, pathophysiologic, or other scientific evidence (Figure 1(d)) [20,21]. In clinical trials, a surrogate endpoint (or marker) is a measure of the effect of a certain treatment that may correlate with a true endpoint but does not necessarily have a guaranteed relationship with it.

According to the Biomarker Working Group [2], a surrogate endpoint is defined as ‘a biomarker intended to substitute for a clinical endpoint. A clinical investigator uses epidemiological, therapeutic, pathophysiological, or other scientific evidence to select a surrogate endpoint that is expected to predict clinical benefit, harm, or lack of benefit or harm.’

The terms ‘biomarker’ and ‘surrogate endpoint’ are often used interchangeably. However, there is a subtle difference. Surrogate endpoints may not merely be biomarkers and could include imaging measurements (such as CT, MRI, and PET); therefore, it is likely that only a few biomarkers would be considered for use as surrogate endpoints. For the concept of a surrogate endpoint to be useful, one must specify the clinical endpoint, class of intervention, and population in which the substitution of the biomarker for a clinical endpoint is considered reasonable [15].

A pharmacodynamic biomarker which correlates well with a widely accepted clinical outcome at both individual and group levels could potentially act as a surrogate endpoint and a substitute for a recognized clinical endpoint, such as the manner in which low density lipoprotein (LDL) cholesterol acts as a surrogate for major cardiovascular events in the licensing of statins [5]. Interested readers can refer to the study published by Fleming and DeMets [22], which provides many examples of surrogate endpoints and which has pointed out that these surrogate endpoints often fail in formal statistical validation.

## 3. Study Designs

In this section, we have reviewed the typical and current study designs that use biomarkers. We have simply assumed that clinical studies involving 2-treatment comparisons (standard treatment versus test treatment) feature one biomarker with two status levels (positive or negative).

### 3.1. Standard Randomized Clinical Trial (RCT) Design

In general, a well-controlled, randomized parallel group design can be useful to identify biomarker(s) in clinical studies that include patients with both high and low values or levels of the biomarker(s). Retrospective analyses of data from these RCTs may be used to identify candidate biomarkers. Buyse *et al.* [11] have discussed the identification and validation of biomarkers.

For a prognostic biomarker, an association must be demonstrated between the value of the biomarker at baseline, or changes in the biomarker over time, and clinical outcome regardless of treatment. For a putative prognostic biomarker to be validated, its association with the clinical endpoint of interest should be demonstrated repeatedly in independent studies, preferably across a range of clinical situations. Retrospective studies may be sufficient for the initial identification and statistical validation of prognostic biomarkers, although the clinical utility or validity of the biomarkers may need to be confirmed in prospective studies.

For a predictive biomarker, the baseline value or changes in the values of the biomarker over time must be shown to predict the efficacy or toxicity of a test treatment, as assessed by a defined clinical outcome. For a putative predictive biomarker to be validated, its ability to predict the effects of treatment (or lack thereof) should be demonstrated repeatedly in multiple studies. Identification of a predictive biomarker requires statistical data from RCTs that include patients with both high and low levels of the biomarker. Retrospective analyses may be sufficient to identify candidate predictive biomarkers; however, prospective research and analyses may be required to validate the biomarkers. The statistical issues for identification of prognostic and predictive biomarkers are discussed in Section 4.

### 3.2. Biomarker by Treatment Interaction Design

**Figure 2.**
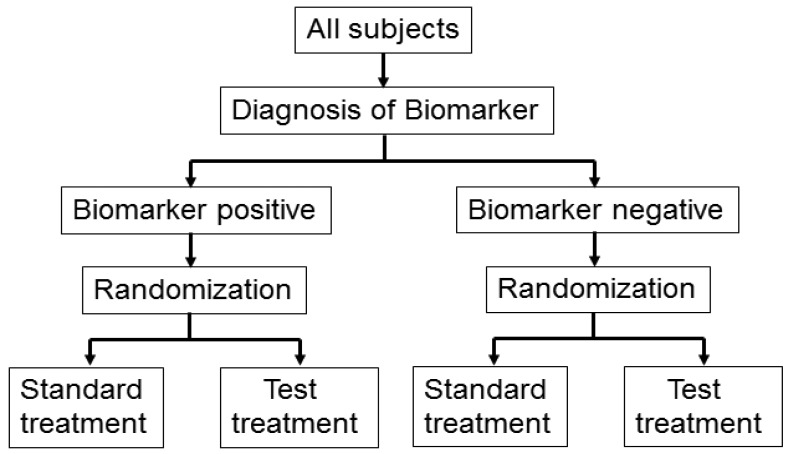
Biomarker by treatment interaction design.

This design is applied to confirm a treatment effect by using biomarker status as a stratification factor (*i.e.*, a population can be divided into biomarker-defined subgroups) [14,23,24]. Patients within each biomarker subgroup are randomly assigned to different treatments (Figure 2). This is similar to conducting 2 independent RCTs to compare different treatments.

An actual application of this design is a Cancer and Leukemia Group B trial (CALGB-30506) to investigate benefit of adjuvant chemotherapy in stage I non-small cell lung cancer (NSCLC) patients [25]. The patients were stratified by their risk group (high or low) defined by a genomic prognostic biomarker [25] and were randomly assigned to either chemotherapy or observation by each risk group.

### 3.3. Biomarker-Strategy Design

Biomarker-strategy design addresses the clinical utility of a biomarker and falls into two classifications: one is biomarker-strategy design with a standard control and the other is that with a randomized control [14,23,24].

**Figure 3.**
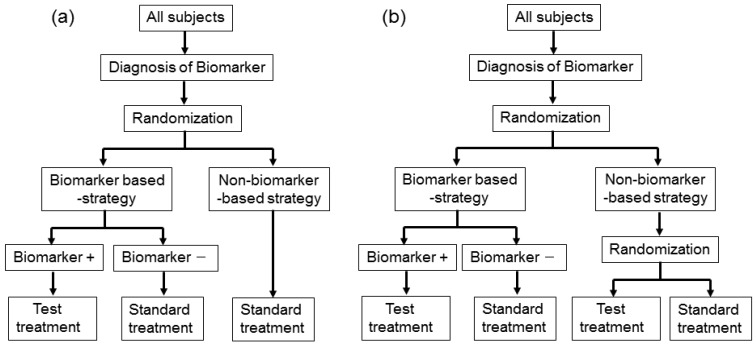
Biomarker-strategy design. (**a**) With standard control and (**b**) with randomized control. ‘+’ and ‘−’ correspond to the respective positive and negative biomarker statuses.

Figure 3 illustrates the summary of this design. First, all patients are randomly assigned to an experimental arm (‘biomarker-based strategy’ arm) that uses the biomarker to determine treatment, or to a control arm (‘non-biomarker-based strategy’ arm) that does not. Next, if the patients allocated to the biomarker-based strategy arm are diagnosed as biomarker positive, they are allowed to proceed to the test treatment. On the other hand, if the patients allocated to the biomarker-based strategy arm are biomarker negative, they then proceed to the standard treatment. In its simplest version, patients in the control arm receive standard treatment, and patients in the experimental arm are treated with either the test or standard treatment, depending on their biomarker statuses (Figure 3(a)). On the other hand, in another version, the patients allocated to the control arm are randomized to standard or test treatments (Figure 3(b)).

However, there is scientific concern regarding this design, as shown in Figure 3. Owing to the potential inability to distinguish between a prognostic effect of the biomarker and the effect of the treatment, this design cannot determine whether differences in outcome result from one or the other of these effects. Further, simulation studies have shown that the two biomarker-strategy designs are generally less efficient than the traditional randomized design [25–30].

Biomarker-strategy design was applied to the GILT docetaxel trial [31] that used DNA excision repair protein (ERCC1) overexpression in tumour RNA (a biomarker of cisplatin resistance) to customize chemotherapy in patients with advanced NSCLC. Patients were randomly assigned in a 1:2 ratio to the control (non-biomarker-based strategy) arm or the genotypic (biomarker-based strategy) arm in which ERCC1 was assessed (Figure 3(a)). Patients in the control arm received a standard regimen of docetaxel plus cisplatin. In the genotypic arm, patients with low ERCC1 levels received docetaxel plus cisplatin, and those with high levels received docetaxel plus gemcitabine [32].

### 3.4. Enrichment Design and Hybrid Design

In actual settings, the potential treatment benefit is limited to a certain biomarker-defined patient subgroup based on sufficiently convincing evidence. Whether or not such evidence exists, there could be a widely held perception that equipoise for the best treatment choice is present only in patients with certain biomarker values. In either case, it is not feasible to use a biomarker-stratified design; therefore, the clinical utility of the biomarker can be partially assessed by an enrichment trial design [33].

**Figure 4.**
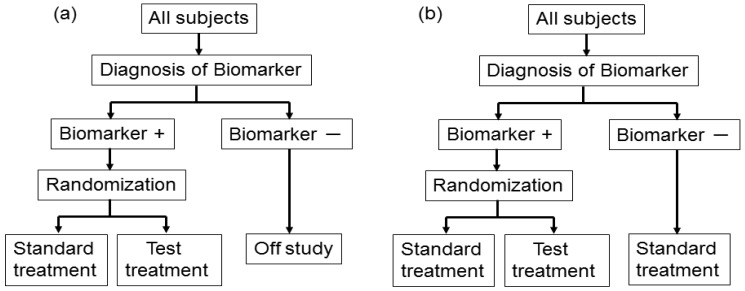
(**a**) Enrichment study design and (**b**) hybrid design. ‘+’ and ‘−’ correspond to the respective positive and negative biomarker statuses.

This design involves a prescreening step whereby patients are selected for the study based on a prespecified biomarker status [34]. First, all patients are screened using a diagnostic test for the biomarker, and only those with positive results are included in the study and randomized to receive either the test treatment or standard treatment. Therefore, this design results in stratification of the study population, with the goal of understanding the efficacy and tolerability of a treatment in the subgroup defined by a prespecified biomarker status (Figure 4(a)). As an actual application, this design was applied to two randomized clinical trials for trastuzumab for breast cancer patients. In these trials, the patients with only human epidermal growth factor receptor 2 (HER2) protein expression-positive metastasis were considered eligible and were randomized [35,36].

As a similar design strategy, Mandrekar and Sargent [23,24] defined a hybrid design in which patients with only a specific biomarker are randomly assigned to treatment based on their biomarker status, whereas patients with the other biomarker are assigned the standard treatment (Figure 4(b)). Although the enrichment design does not provide data on the effectiveness of the standard treatment for biomarker-negative patients, this is one of the optimal designs when compelling prior evidence demonstrating the efficacy of a certain treatment for a biomarker subgroup renders it unethical to randomly assign patients with that particular biomarker status to other treatment options.

In fact, this design was applied to a clinical trial to evaluate a prognostic and possibly predictive biomarker for breast cancer, Oncotype Dx^®^, in breast cancer patients treated with tamoxifen [37–41]. This study was the first to test the feasibility of a prognostic biomarker in a clinical application. This trial is an example of prospective validation trials utilizing a hybrid design that has the potential to substantially change patient management in the future, allowing for better risk assessment and improved individualized treatment.

### 3.5. Adaptive Signature Design

**Figure 5.**
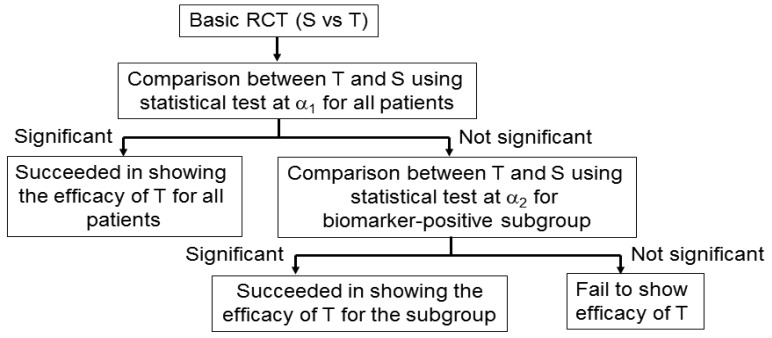
General procedure for adaptive signature and biomarker-adaptive threshold designs. ‘S’ and ‘T’ denote the standard and test treatments, respectively.

An adaptive signature design is a 2-stage design for randomized clinical trials of targeted agents in settings where an assay or signature that identifies sensitive patients is not available at the outset of the study [42] (Figure 5). In the first stage of this design, the clinical outcome of test treatment is compared to that of standard treatment for all randomly allocated patients in the study by the application of a statistical test at significance level *α*_1_. If the effect of the test treatment is superior to that of the standard treatment for statistically significant, analysis is done. If there is no significant difference between the two treatments, testers proceed to the second stage. In the second stage, the predictive biomarker for a subset of patients, *N*_1_ (the number of patients of the subset), in the study is identified by a statistical classification method. Next, patients positive for the identified biomarker are defined from the remaining subset of patients, *N*_2_ (= total sample size *N* − *N*_1_), and the outcome of the test treatment is compared with that of the standard treatment for the specified biomarker-positive patients with the statistical test at significance level *α*_2_.

This design includes the multiplicity problem for statistical testing since the statistical test would be conducted twice. Hence, to control the overall significance level to that under a nominal value *α* (typically, *α* = 0.05), we must specify the significance level as *α* = *α*_1_ +*α*_2_. This multiplicity problem is discussed in detail in Section 4. In addition, Freidlin and Simon [42] recommended that *α*_1_ = 0.04, *α*_2_ = 0.01, and that the ratio of *N*_1_ to *N*_2_ be 1. Furthermore, the procedure to identify the predictive biomarker should be prespecified if this design is to be applied.

Freidlin and Simon [42] investigated the statistical power of the adaptive signature design and standard RCT. They determined that the power of the adaptive signature design was higher than that of the RCT when there is a predictive biomarker. The power loss of the adaptive signature design was not critical even when there was no predictive biomarker. The adaptive signature design would be a practical approach when evidence about the predictive biomarker is insufficient before conducting the clinical study. Recently, Freidlin *et al.* [43] proposed an improved adaptive signature design named the ‘cross-validated adaptive signature design’.

### 3.6. Biomarker-Adaptive Threshold Design

Jiang *et al.* [44] proposed a statistically rigorous biomarker-adaptive threshold phase III design to develop a biomarker which is measured on a continuous or graded scale to identify patients who are sensitive to a test treatment (Figure 5). In general, the main purpose of this design was to identify and validate a cut-off point for a prespecified biomarker, and to compare the clinical outcome between test and standard treatments for all patients and for the patients identified as biomarker positive in a single study. The procedure provides a prospective statistical test of the hypotheses that the test treatment is beneficial for the entire patient population or that the test treatment is beneficial for a subgroup defined by the biomarker, and provides an estimate of the optimal biomarker cut-off point. With this design, human samples such as blood or tumour specimens are collected to measure a prespecified biomarker from all patients at the time of enrolment, but the biomarker is not used as an eligibility criterion. This strategy of combining two statistical tests can be applied in the following two analysis plans: First, a test for comparison between test and standard treatments in all patients is conducted at a prespecified significance level, *α*_1_. If the test is statistically significant, the procedure is done, and the outcome difference between the two treatments for all patients is confirmed. If the test is not significant, second-stage analysis is conducted to identify an optimal cut-off point for the predictive biomarker using the remaining significance level (*α*_2_ = *α* − *α*_1_). This procedure (hereafter referred to as ‘analysis plan A’) controls the probability of making any false-positive findings at the prespecified level, *α*. To preserve the ability of analysis plan A to detect an overall effect, Jiang *et al.* [44] recommended setting *α*_1_ = 0.04 and *α*_2_ = 0.01 to correspond to an overall significance level, *α* = 0.05. The advantage of analysis plan A is its simplicity and that it explicitly separates the effect of the test treatment in the broad population from the subgroup specification. However, the analysis plan takes a conservative approach in adjusting for multiplicity in combining the overall and subgroup analyses.

Otherwise, ‘analysis plan B’ combines the two statistical tests for overall and subgroup patients by incorporating the correlation structure of the two test statistics, and is a generalization of analysis plan A. For example, if analysis plan B demonstrates a difference between the test and standard treatments by the statistical test, the next step is to identify the biomarker threshold above which the test treatment is more effective than the standard treatment.

Additionally, in both analysis plans A and B, a point estimate and a confidence interval for the cut-off point are estimated by using a bootstrap re-sampling approach. However, the cut-off value should not be estimated if analysis plan B does not demonstrate a statistical difference between the two treatments, as the estimation is inexplicable [44]. Furthermore, a simulation study comparing the performance between analysis plan A and analysis plan B found that the latter was more effective than the former, but that both were superior to the overall test ignoring the biomarker in cases where less than half of the patients benefited from the test treatment [44].

### 3.7. Adaptive Accrual Design

**Figure 6.**
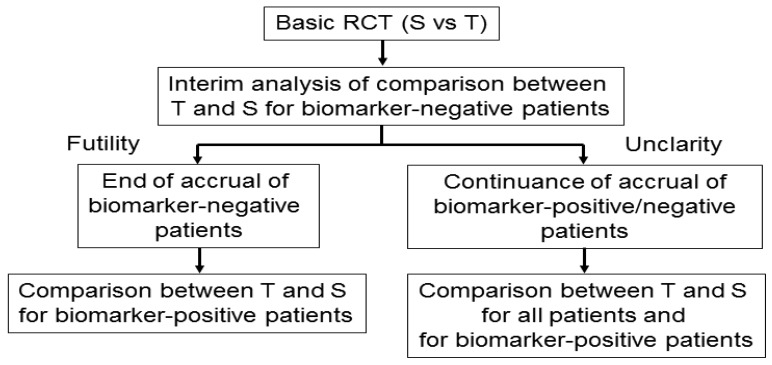
General procedure for adaptive accrual design. ‘S’ and ‘T’ denote the standard and test treatments, respectively.

Wang *et al.* [45] proposed an adaptive accrual phase III design comparing a test treatment with a standard treatment that begins with accruing both biomarker-positive and biomarker-negative patients (Figure 6). This design modifies accrual adaptively to two prespecified biomarker subgroups based on an interim futility analysis; therefore, an interim analysis is performed to evaluate the test treatment in the biomarker-negative patients. If the interim analysis indicates that confirming the effectiveness of the test treatment for the biomarker-negative patients is futile, then the accrual of biomarker-negative patients is halted and the final analysis is restricted to evaluating the test treatment for the biomarker-positive patients. Otherwise, accrual of biomarker-negative and biomarker-positive patients continues to the target sample size until the end of the trial. At that time, the test treatment is compared to the standard treatment for the overall population and for biomarker-positive patients.

According to a simulation study investigating the performance characteristic of this design in Wang *et al.* [45], this design demonstrated a greater power than a standard RCT design (non-adaptive trial); however, the design accrues many more biomarker-positive patients and may require a much longer trial duration depending on the prevalence of the biomarker. In addition, the futility boundary is somewhat conservative and less than optimal as it is set to be in the region where the observed efficacy for the standard treatment is greater than that for the test treatment.

### 3.8. Bayesian Adaptive Design

**Figure 7.**
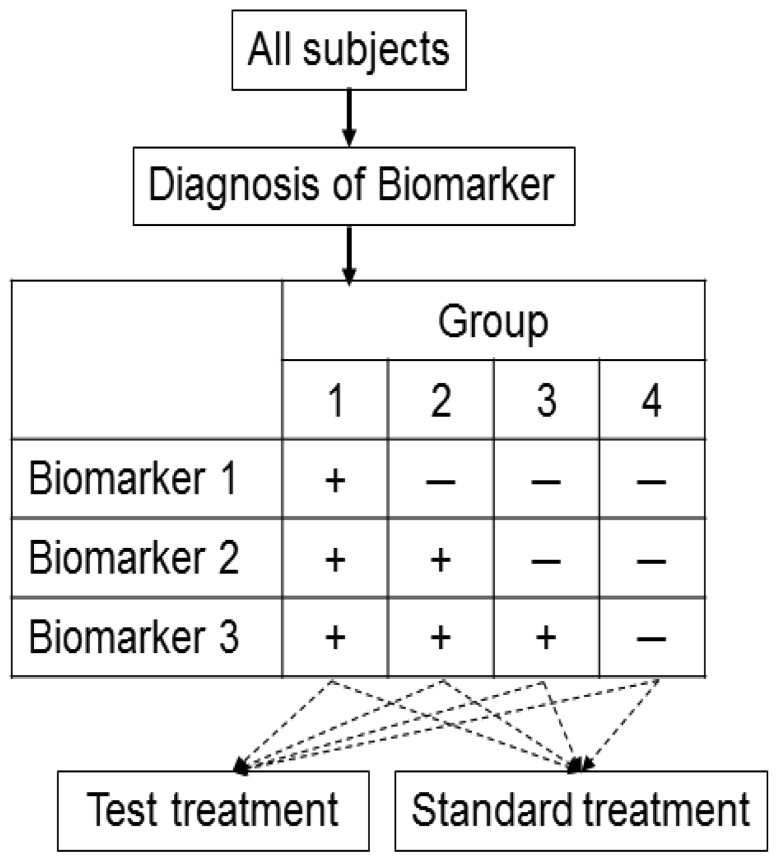
Framework for Bayesian adaptive design. Patients are assigned to biomarker groups 1–4 in sequential order according to the characteristics of the three biomarker categories. ‘+’ and ‘−’ correspond to the respective positive and negative biomarker statuses. Patients are adaptively randomized to one of the three treatments according to their biomarker groups. The dashed arrows indicate the putative effective treatment for each of the biomarker groups.

Zhou *et al.* [46] proposed an outcome-based adaptive randomization design for targeted treatment using a Bayesian hierarchical framework to randomly assign patients to treatments based on their biomarker status (Figure 7). The Bayesian hierarchical probit model [47] is used to characterize a clinical outcome for each treatment in a biomarker-defined subgroup. Bayesian adaptive design can help to refine the estimation and randomization of the patients as the trial progresses. The randomization rate is then computed based on the estimated posterior mean of the clinical outcome of each treatment in each biomarker subgroup. Adaptive randomization will be performed until the last patient is enrolled, unless the trial is ended early because all treatments are suspended due to futility. For each biomarker status, better performing groups will have higher randomization rates. Early stoppage rules are set so that low-performing groups may be suspended for new patients to be randomized into the groups.

As an application setting, this design has been used for evaluation of the phase II Biomarker-Integrated Approaches of Targeted Therapy of Lung Cancer Elimination (BATTLE) trial, which consists of an umbrella screening trial and four parallel phase II targeted therapy trials (adaptively randomized into one of the four treatments using erlotinib, sorafenib, vandetanib, and the combination of erlotinib and bexarotene) in advanced NSCLC patients with prior chemotherapy.

Bayesian statistical methods are being used increasingly in clinical research because the Bayesian approach is ideally suited to adapting to information that accrues during a trial, potentially allowing for smaller more informative trials and for patients to receive better treatment. Bayesian design can provide an advantage over the non-Bayesian if certain conditions exist and have been the topic of a recent FDA guidance publication [48]. However, Zhou *et al.* [46] has pointed out some limitations for the use of Bayesian adaptive designs and the designs are not necessarily more efficient non-Bayesian designs [49]. As for choosing beforehand the type of analysis to be used Bayesian or non-Bayesian, we recommend it should be carefully considered and determined based on the research purpose and the prior information for related studies.

## 4. Statistical Issues for Biomarker Studies

In this section, we introduce the important issues of confounding and multiplicity that arise quite often in biomarker studies.

### 4.1. Confounding and Interaction

In clinical studies to evaluate the treatment effect, many sources of variation have an impact on the evaluation of the treatment. If these variations are not identified and properly controlled, then they may be combined with the treatment effect that the studies are intended to demonstrate. In this case, the treatment is said to be confounded with the effects due to these variations [50].

To provide a better understanding, consider the following example. A test treatment was compared to a standard treatment for a clinical outcome in an RCT. All patients allocated to the two treatment groups in the study were biomarker negative. As a result, the outcome with the test treatment was not superior to the standard treatment, and the test treatment effect for those patients was not confirmed. However, as mentioned earlier, all patients were biomarker negative. Thus, it is not clear whether the insufficient treatment effect was due to the use of the test treatment or the effect of being biomarker negative. In this example, the biomarker is termed a potential confounding factor of the treatment effect.

Further, the laboratory batch effects due to assay runs, reagent lots, and shifts in instrument calibration often pose significant risks for confounding. For instance, we consider a study in which blood samples from test treatment were treated with Reagent I and blood samples from standard treatment were treated with Reagent II. If the reagent effect was associated with the outcome, the reagent was a confounding variable; a confounding variable is a variable that is associated with the treatment and the outcome. Therefore, the study cannot separate effects of the confounding variable and the treatment effect.

Close contemplation at the planning stages of a biomarker study is very important to avoid this issue. Randomization and selection of the study population (such as inclusion and exclusion criteria) can be useful tools to prevent confounding. In the statistical analysis stage, removal of the confounding effect is also attempted in order to perform subgroup analysis and model-based analysis, which are typical approaches in biomarker studies.

#### 4.1.1. Subgroup Analysis

A subgroup analysis is the simplest approach and an important part of the analyses in a comparative clinical study. Separate comparison of the test treatment to the standard treatment would be conducted in the subgroups (e.g., biomarker positive and biomarker negative) for a specific clinical outcome. When multiple subgroup analyses are performed, the results are commonly over-interpreted and can lead to further research that is misguided, or worse, lead to suboptimal patient care due to substantial inflation of the probability of a false positive result [51]. This issue constitutes multiplicity for statistical testing (discussed in Section 4.2).

In cases where, *a priori*, one does not expect the treatment to be effective in the biomarker-negative patients unless it is effective in the biomarker-positive patients, one might structure the analysis in the following manner: compare the test treatment to the standard treatment in the biomarker-positive patients using a significance threshold of 0.05. If the treatment difference for the biomarker-positive patients is not significant, do not perform the statistical significance test in the biomarker-negative patients. Otherwise, compare the treatment to the control in the biomarker-negative patients using a statistical significance threshold of 0.05. This sequential approach controls the overall false positive rate (Type I error) at 0.05 [8].

#### 4.1.2. Model-Based Analysis

The traditional statistical approach in which cases are classified by treatment and by a biomarker as a covariate that may affect treatment efficacy is to first test whether there is a significant interaction between the treatment (test versus standard treatment) and the covariate (biomarker negative or biomarker positive). If the interaction test is not significant, then the treatment effect can be evaluated overall, and not within the levels of the biomarker. If the interaction test is significant, the biomarker may be regarded as a predictive biomarker. The treatment effect differs with each biomarker status and is evaluated separately within the levels of the biomarker (Figure 1(b)). Therefore, a predictive biomarker is generally identified by the interaction test between the treatment and biomarker, where the interaction may be either qualitative or quantitative.

In practice, the interaction test is often performed based on the use of statistical models, which are an integral component of any data analysis describing the relationship between clinical outcome and one or more explanatory variables (such as treatment group and biomarker status) in the form of mathematical equations. For example, an RCT was conducted to identify a predictive biomarker for progression-free survival. The Cox proportional hazard model [52] is often applied to the survival time and formulation of the model as follows:

(1) $$\text{Hazard}\;=h_{0}\exp(b_{1}\;\text{Treat}\;+b_{2}\;\text{Bio}\;+b_{3}\;\text{Treat}\times \text{Bio})$$

where *h*_0_ is a baseline hazard as a nuisance parameter, and *b*_1_ and *b*_2_ are the effects of treatment and biomarker status, respectively. An interaction effect by treatment and biomarker status is *b*_3_. Treat = 0, 1 indicates whether the patients were allocated standard or test treatments, respectively; Bio = 0, 1 denotes negative or positive biomarker status, respectively. Treat × Bio indicates the interaction term. To identify whether the biomarker is predictive, the interaction effect *b*_3_ is statistically tested. In addition, the hazard ratio (HR) is a useful statistic for quantitative interpretation of the treatment effect and interaction effect. In particular, the HR of the test treatment to the standard treatment in biomarker-negative patients is estimated as exp(*b*_1_), and the HR for biomarker-positive patients is estimated as exp(*b*_1_ + *b*_3_), as derived from Equation (1). Additionally, a logistic model for a binary outcome and analysis of (co)variance (ANOVA or ANCOVA) model for a continuous outcome are often applied to evaluate the interaction effect by treatment and biomarker status.

Statistical tests of interaction effect should be used instead of inspection of subgroup *p*-values, which often encourages inappropriate subgroup claims. Only if the statistical interaction test supports a subgroup effect should the conclusions be influenced. Even then, the emphasis should depend on biological plausibility, the number of subgroup analyses, their prespecifications, and the statistical strength of evidence to acknowledge that most subgroup claims are prone to exaggerating the truth [53].

### 4.2. Multiplicity

Simultaneous considerations of a set of statistical inferences are common in clinical studies. Clinical studies frequently incorporate one or more of the following design features: multiple outcome measures, repeated tests of significance as the study progresses (interim analyses) to ensure early detection of effective treatments, subgroup analysis to address particular concerns on the efficacy and safety of the drug in specific patient subgroups (e.g., biomarker positive/negative), and various combinations of these features.

Multiplicity is an important issue for multiple testing in the planning, data analysis, and interpretation of clinical studies. In this section, we first introduce the framework and principle of statistical tests. Next, we present the multiplicity issue of multiple testing.

#### 4.2.1. Statistical Test Procedure

A ‘statistical hypothesis test’ is a formal scientific method to examine the plausibility of a specific statement regarding the comparison of an outcome between one group and a fixed value or between two or more groups. We adopt the 2-treatment group comparison in this section.

The statement regarding the comparison is typically formulated as a ‘null hypothesis’ stating that there is no difference in outcome between the test and standard treatments. An ‘alternative hypothesis’ is set for the study objective to be proved, such as that the mean of the outcome between the two treatments will differ. The test procedure computes a *p*-value based on the null hypothesis to summarize the test result. The *p*-value is the probability of obtaining an experimental outcome as observed or that which is more extreme if the null hypothesis is true.

When a statistical test is performed, one of four outcomes will occur, depending on whether the null hypothesis is true or false and whether the statistical test rejects or does not reject the null hypothesis: (1) the procedure rejects a true null hypothesis (a false-positive type I error), (2) the procedure does not reject a true null hypothesis (a true negative), (3) the procedure rejects a false null hypothesis (a true positive), or (4) the procedure does not reject a false null hypothesis (a false-negative type II error). The true state and the decision to accept or reject a null hypothesis are summarized in Table 2 [54,55].

#### 4.2.2. Multiplicity of a Statistical Test

Multiplicity is an important issue for inflating Type I error when multiple simultaneous hypotheses are tested at set *p*-values. When more than 1 test is used, the chance of finding at least 1 test statistically significant due to chance and incorrectly declaring a difference increases. When 10 statistically independent tests are performed, each with a significance level of 0.05, the chance of at least 1 test being significant is no longer 0.05, but approximately 0.40 = 1 − (1 − 0.05)^10^. This phenomenon is termed a Type I error inflation and multiplicity problem [56]. To accommodate the issue, even if more than 1 test is done, it can be necessary to control the overall Type I error to less than the threshold of 0.05 in a clinical study, in particular, in the case of a confirmatory clinical study.

In practical settings, multiplicity would arise in the following situations: testing for multiple endpoints; exploration of multiple prognostic biomarkers; comparison between more than two treatment groups; adaptive design (see Section 3) and interim analyses; basic subgroup analyses; and so on.

The basic ideas of multiple testing have been outlined and the problem of how to control the Type I error has been discussed [55,57]. Bauer [57] mainly emphasized the concept of multiple levels of significance (controlling the study whole or familywise error in the strong sense) which can be achieved by applying the principle of closed tests. Tests based on global statistics, the union intersection principle, and other criteria are discussed.

One of the simplest approaches to account for multiplicity is to adjust the significance level to account for the number of tests [54]. This is achieved by dividing the significance level for each test by the number of tests performed, the so-called Bonferroni correction.

For pharmacogenomic studies and genome-wide association studies (GWAS) that focus on finding sets of predictive genes, an alternative approach to multiple testing considers the false discovery rate (FDR), which is the probability that a given gene identified as differentially expressed is a false positive. The FDR is typically computed after a list of differentially expressed genes has been generated [58]. Unlike a significance level, which is determined before looking at the data, FDR is a post-data measure of confidence. It uses information available in the data to estimate the proportion of false-positive results that have occurred.

We cannot introduce other methods for multiplicity adjustment in detail because a large number of methods have been proposed. The interested reader can refer to the papers published by Bauer [57], Westfall and Young [59], Godfrey [60], Hochberg and Tamhane [61], Miller [62], and Tamhane [63].

### 4.3. Statistical and Clinical Significances

Statistical tests are one of the most popular tools, not only in clinical trials but also scientific research, however, many researchers have a confused interpretation about *p*-value and they misuse a number of *p*-values. Statistical significance and clinical significance are not the same thing. The magnitude and direction of the effect must be considered. Thus, confidence interval is more helpful than statistical test to assess the presence or absence of the clinical significance. Even if there are many results that are statistically significant—not likely due to the play of chance, they may be irrelevant due to the small clinical effect. Conversely, a lack of statistical significance should not be confused with a negative result; it may arise from a lack of statistical power due to a limited sample size.

In general, statistical significance is meaningful in confirmatory trials, but not necessary in exploratory trials. In contrast to confirmatory trials, the objectives of exploratory trials may not always lead to simple statistical tests of pre-defined hypotheses [64].

## 5. Summary

The importance of biomarkers in medical diagnosis, prevention, and therapy of diseases is increasing. In fact, studies have identified an impressive number of clinical biomarkers. This article provides an overview on the study designs for biomarker research. In addition, we introduce confounding and multiplicity of statistical tests, which are important statistical issues in biomarker studies. From the viewpoint of evidence-based medicine, appropriate study design and statistical analysis are absolutely necessary for conducting valid biomarker studies.

## Figures and Tables

**Table 1.** Examples of biomarker use.

**Biomarker**	**Current Use**	**Classification**
Human epidermal growth factor receptor 2 (HER2), epidermal growth factor receptor (EGFR), V-Ki-ras2 Kirsten rat sarcoma viral oncogene homolog (KRAS) mutations [3,5,6,8]	Directing treatment	Predictive biomarker
BCR-ABL (*i.e.*, Philadelphia chromosome in CML) [6]	Directing treatment of imatinib	Predictive biomarker
Cytochrome P450 enzymes (CYP2D6, CYP2C9, CYP2C19 polymorphisms) [3,5,7]	Known to affect drug metabolism	Predictive biomarker
Estrogen receptor (ER) and progesterone receptor (PR) [4,6]	Selection for hormonal therapy	Predictive biomarker
Promyelocytic leukemia-retinoic acid receptor α (PML/RARα translocation [9]	Prescribing arsenic trioxide for acute promyelocytic leukaemia	Predictive biomarker
Uridine diphosphate glucuronosyltransferase (UGT1A1), Thiopurine Methyltransferase (TMPT), major histocompatibility complex, class I, B (HLA-B*5701), Dihydropyrimidine dehydrogenase (DPYD) polymorphisms) [5]	Predisposition to certain toxicities	Predictive biomarker
Amyloid β peptide (AB) 1-42 [3,5]	Diagnosis of prodromal Alzheimer's disease	Prognostic biomarker
Gene signature chips (e.g., Oncotype, MammaPrint) [5]	Prognosis prediction in oncology	Prognostic biomarker (also predictive in certain cases)
B-type natriuretic peptide (BNP) [10]	Screening and diagnosis in heart failure	Prognostic biomarker
C-reactive protein (CRP), Interleukin-6 (IL-6), Tumor necrosis factor (TNF-α in blood samples [3,5]	Proof of principle in inflammatory diseases	Pharmacodynamic biomarker
FDG-PET (SUVmax) functional imaging [3,5]	Proof of concept (e.g., in tumour metabolism)	Pharmacodynamic biomarker
Low density lipoprotein (LDL) cholesterol [5]	Confirmatory trials in coronary heart disease	Surrogate endpoint
Hemoglobin a1c (HbA1c) [5]	Represents glycaemic control in diabetics	Surrogate endpoint
Prostate-specific antigen (PSA) [6]	Screening and monitoring in prostate cancer	Surrogate endpoint
Carcinoembryonic antigen (CEA) and cancer antigen (e.g., CA-19-9) [4,6]	Monitoring in cancers	Surrogate endpoint

**Table 2.** True state and hypothesis test.

	**Hypothesis Test (Is the Null Hypothesis Rejected?)**	
**True State (there is No Difference between the 2 Groups)**	Yes	No
Yes	(1) False positive (Type I error)	(2) True negative
No	(3) True positive (Power)	(4) False negative (Type II error)
